# Full-Genome Sequence of the Plant Growth-Promoting Bacterium *Pseudomonas protegens* CHA0

**DOI:** 10.1128/genomeA.00322-14

**Published:** 2014-04-24

**Authors:** Alexandre Jousset, Joerg Schuldes, Christoph Keel, Monika Maurhofer, Rolf Daniel, Stefan Scheu, Andrea Thuermer

**Affiliations:** a Institute of Environmental Biology, Utrecht University, Utrecht, The Netherlands; b Institute of Microbiology and Genetics, Department of Genomic and Applied Microbiology & Göttingen Genomics Laboratory, Georg August University Göttingen, Göttingen, Germany; c Department of Fundamental Microbiology, University of Lausanne, Lausanne, Switzerland; d Plant Pathology, Institute of Integrative Biology, Swiss Federal Institute of Technology (ETH), Zürich, Switzerland; e JFB Institute of Zoology and Anthropology, Georg August University Göttingen, Göttingen, Germany

## Abstract

We report the complete genome sequence of the free-living bacterium *Pseudomonas protegens* (formerly *Pseudomonas fluorescens*) CHA0, a model organism used in plant-microbe interactions, biological control of phytopathogens, and bacterial genetics.

## GENOME ANNOUNCEMENT

*Pseudomonas protegens*, formerly part of the *Pseudomonas fluorescens* complex (1), is a free-living eubacterium isolated from tobacco roots in Switzerland (2). It produces secondary metabolites with broad-spectrum antibiotic activity (hydrogen cyanide [HCN], 2,4-diacetylphloroglucinol, pyoluteorin, and pyrrolnitrin) associated with the inhibition of phytopathogens (3), as well as the insecticidal toxin Fit (4). Like *P. protegens* Pf-5, strain CHA0 has reduced catabolic potential compared to other fluorescent pseudomonads, indicating that *P. protegens* may have specialized in plant-derived exudates.

The genome of CHA0 was sequenced with a combination of 454 sequencing and Illumina platforms. Gaps and repetitive regions were resolved with Sanger sequencing. The 6.87-Mbp genome has 63.4% G+C content and contains five rRNA operons and 68 tRNAs. It contains three complete prophages and seven candidate clustered regularly interspaced short palindromic repeat (CRISPR) clusters, suggesting that interactions with phages contributed to shaping the genome. It encodes only one predicted functional bacteriocin, which is in line with its relatively low antagonistic activity against other pseudomonads (5). This bacterium possesses important traits linked to plant colonization and protection, including the production of siderophores and exoenzymes (e.g., proteases, chitinases, and phospholipases). It possesses a type VI secretion system.

The genome is very close to that of *P. protegens* Pf-5 (98.87% of the total aligned nucleotides), confirming that *P. protegens* is an independent taxon well separated from other pseudomonads (1).

### Nucleotide sequence accession number.

The nucleotide sequence is deposited in NCBI under the accession no. CP003190.
